# Fully Integrated Line Array Angular Displacement Sensing Chip

**DOI:** 10.3390/s23052431

**Published:** 2023-02-22

**Authors:** Yunhao Fu, Jiaqi Jiang, Zhuang Zhao, Zhongyuan Zhao, Kaixin Chen, Min Tao, Yuchun Chang, Guoqiang Lo, Junfeng Song

**Affiliations:** 1 State Key Laboratory of Integrated Optoelectronics, College of Electronic Science and Engineering, Jilin University, Changchun 130012, China; 2 Faw Jiefang Group Co., Ltd., Changchun 130012, China; 3 Northeast Electric Power Design Institute Co., Ltd. of China Power Engineering Consulting Group, Changchun 130000, China; 4 School of Microelectronics, Dalian University of Technology, Dalian 116620, China; 5 Advance Micro Foundry Pte. Ltd., Singapore 117685, Singapore; 6 Peng Cheng Laboratory, Shenzhen 518000, China

**Keywords:** angular displacement sensing chip, integrated optoelectronic chip, pseudo-random code, incremental code, SAR ADC, CMOS process

## Abstract

The angular displacement sensor is a digital angular displacement measurement device that integrates optics, mechanics, and electronics. It has important applications in communication, servo control, aerospace, and other fields. Although conventional angular displacement sensors can achieve extremely high measurement accuracy and resolution, they cannot be integrated because complex signal processing circuitry is required at the photoelectric receiver, which limits their suitability for robotics and automotive applications. The design of a fully integrated line array angular displacement-sensing chip is presented for the first time using a combination of pseudo-random and incremental code channel designs. Based on the charge redistribution principle, a fully differential 12-bit, 1 MSPS sampling rate successive approximation analog-to-digital converter (SAR ADC) is designed for quantization and subdivision of the incremental code channel output signal. The design is verified with a 0.35 μm CMOS process and the area of the overall system is 3.5 × 1.8 mm^2^. The fully integrated design of the detector array and readout circuit is realized for the angular displacement sensing.

## 1. Introduction

The angular displacement sensor is a precise device combining numerous technologies, including circuitry, mechanical, and optical components. To measure angular velocity and other parameters, the sensor converts angular displacement to digital or pulse electrical signals. The angular displacement sensor is widely used in high-precision measurement applications for manufacturing, aircraft, and other industries owing to its high accuracy, high resolution, contactless measurement, and ease of downsizing [1,2,3]. Due to advancements in the semiconductor process technology and the theoretical underpinnings of Moiré stripes, angular displacement sensors used in these fields typically process architectures based on optical, capacitive, and inductive principles [4,5,6]. Compared to other types of angular displacement sensors, optical encoders realize absolute measurements by encoding optical signals into pseudo-random or binary codes, which are characterized by high accuracy, high reliability, and small size [7,8].

Based on the variations in their operating principles and coding methodologies, optical encoders can be classified into incremental, absolute, and hybrid encoders. The incremental encoders use the output signal in determining the relative position; however, there are a few drawbacks, such as cumulative errors and data loss during power failure. In contrast, absolute encoders use digital coding for extracting absolute position information; nevertheless, the significant amount of coding effort that is required makes it challenging to create downsized designs [9,10,11]. Furthermore, the hybrid coding technique maximizes measurement resolution while maintaining miniaturization by combining the benefits of incremental and absolute code channels [12,13].

The high-precision scribing code plate and indicator grating used in the traditional optical shaft angle encoder’s system implementation serve as the detection device. To measure displacement, a circuit translates, amplifies, and divides the optical signal into a Moiré stripe signal after traveling via a code plate and an indicator grating. Despite the fact that traditional angular displacement sensors have been able to achieve high measurement accuracy and resolution, the size of such high performance optical encoders has the limitation of being too large for some applications, such as for automotive [14,15]. This is because the encoder frequently requires complex grating disk scribing, due to its optical and electrical principles, and the design of the optical receiver makes it impossible to achieve a high degree of integration.

Previously, an absolute encoder [16] was designed based on an area array image sensor and obtained a level of 3.2 k frame rate and 14-bit resolution. In 2017, Wu et al. proposed a method by applying pseudo-random code channels to slit disk and using a linear CCD sensor as a photodetector, which utilizes the space of slit disk [17]. Consequently, small size disk high resolution coding was achieved. In [18], a line array based absolute encoder was designed to obtain a 22-bit resolution with 5.6″ interpolation accuracy. In [19], a self-correction error compensation method of three image sensors was applied to the angular displacement system. The study of [13] proposed a photodiode compensation method applied to an optoelectronic encoder chip. This method compensates the area of the photodiode array according to the light intensity distribution characteristics of the light source. Furthermore, a photodiode array chip is designed for reflective hybrid optoelectronic encoder based on the CMOS process to achieve high consistency of the output signal.

In this study, a hybrid pseudo-random code channel, incremental code channel, and on-chip integrated successive approximation analog-to-digital converter (SAR ADC) were used to design an absolute angular displacement-sensing chip. The primary benefit of the hybrid coding method is that absolute zero position and incremental cumulative error problems can be avoided with pseudo-random coding. More importantly, the on-chip integrated SAR ADC module accomplishes the objective of shrinking and monolithic integration of the high-precision angular displacement sensor, which significantly enhances the integration of the system while guaranteeing measurement accuracy.

## 2. Optoelectronic Chip Design

### 2.1. System Architecture

**Figure 1:**
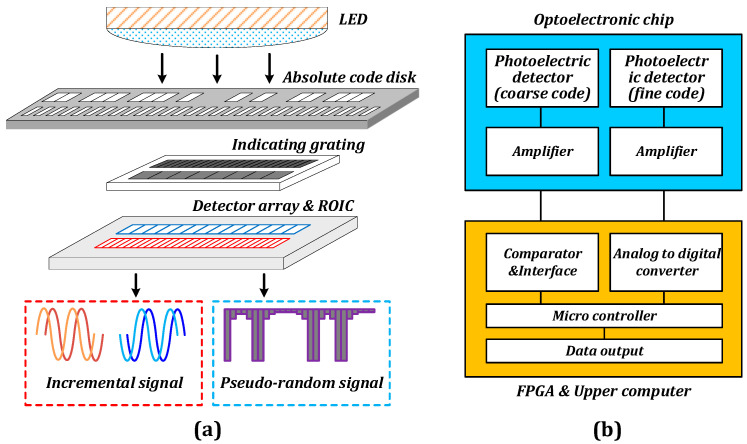
(**a**) Principle of angular displacement reading. (**b**) Mixed-channel optical encoder chip architecture.

The incremental code channel based on the subdivision of the Moiré signal is utilized as the fine code in the angular displacement sensing system based on hybrid coding, whereas the pseudo-random code channel is generally employed as the coarse code. The absolute position readout method is depicted in Figure 1a [20], which involves illuminating the main grating with a light-emitting diode (LED) light source, receiving the optical signal via the code plate and grating, and performing photoelectric conversion on the signal. Subsequently, the amplified signal is outputted by the readout circuit corresponding to the two code channels.

Figure 1b depicts the architecture of a typical photoelectric encoder chip [21]. The peripheral system primarily consists of a comparative amplitude discriminating circuit, analog-to-digital converter, micro-control system, interface circuit, power supply, and several other components. The analog-to-digital converter quantizes the output analog signal of the fine code channel, which is then integrated with the coarse code channel data by the microcontroller to provide the angular displacement information. Additionally, the signal output from the fine code channel is affected by the noise incurred during the signal transfer process, which necessitates the use of additional hardware resources to compensate the signal in a high-precision angular displacement system because the detector, readout circuit, and analog-to-digital converter circuit are not integrated in the optoelectronic chip.

**Figure 2:**
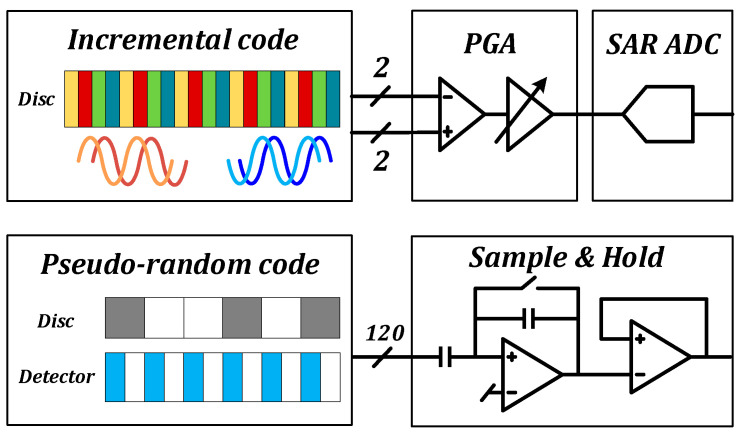
Proposed fully integrated optoelectronic chip. (In the code disc diagram, different colors represent different detection units. In the pseudo-random code channel, the white area and the gray area indicate different shading states).

The proposed fully integrated optoelectronic chip displayed in Figure 2 utilizes a hybrid coding technique that combines the benefits of incremental and absolute coding. It is a simple structure, being stable, reliable, and can also successfully address the problems of error accumulation and zero-position loss during power down and power up. A 12-bit SAR ADC was developed and monolithically combined into the detector and readout circuit to reduce the peripheral hardware overhead of the optical coding system.

### 2.2. Mixed Coding Method

#### 2.2.1. Pseudo-Random Coding Principle and M-Sequence Generation

The coding of absolute positions is achieved by utilizing pseudo-random coding theory using M-sequences, which are deterministic sequences with random characteristics that are predictable and repeatable and entirely composed of binary code. The elements of the M-sequence are uncorrelated with one another and have strong anti-interference and equilibrium properties. Moreover, M-sequences are extremely effective at encoding and have the longest period under the same conditions compared to other pseudo-random sequences.

Generally, M-sequences are generated by linear feedback shift registers, and for an N-stage linear feedback shift register, the maximum period of the resulting sequence is 2^N − 1^, which is an M-sequence. The cyclic units in the above M-sequence can be translated to pseudo-random absolute position codes by mapping each “0” or “1” to a segment of equal length of transmissive or opaque medium.

**Figure 3:**
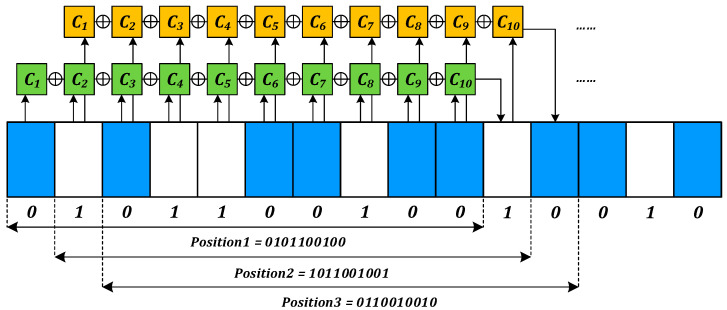
Principle of absolute position coding.

Figure 3 illustrates the absolute position coding of the angular displacement sensing system [22]. The white square corresponds to the translucent area, coded as “1,” and the blue square corresponds to the opaque area, coded as “0”. Each 10-bit code represents a set of pseudo-random codes corresponding to the address data. Assuming that the initial position code consists of “0101100100,” the I-position code can be expressed as

(1) $$X_{i}{=c}_{1}X_{i\;-\;1}c_{2}X_{i\;-\;n+1}⊕\cdot \cdot \cdot c_{n}X_{i\;-\;1}$$

where ⊕ symbolizes the heterogeneous operation, c_1_–c_n_ represents the coefficient of the n-bit code operation, and up to 2n different combinations of codes can be generated by changing c_1_–c_n_. The line array sensor for the pseudo-random channel “takes photo” of the current position data as the code disk rotates, and the readout circuit recognizes and decodes the collected data to determine the absolute position.

#### 2.2.2. Disc

**Figure 4:**
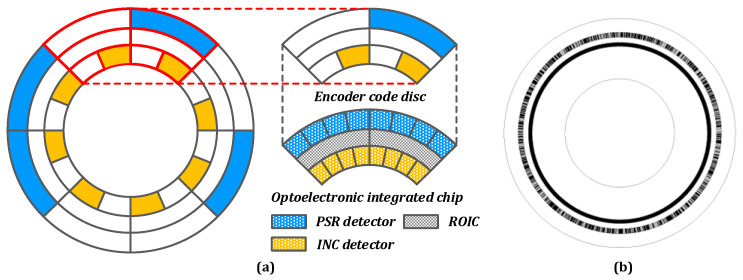
(**a**) Simplified schematic diagram of the hybrid coding code disc with the optoelectronic integrated chip. (The white area corresponds to the translucent area, and both blue area and yellow area corresponds to the opaque area.) (**b**) Diagram of the code disc.

A simplified schematic of the code disk and optoelectronic integrated chip is illustrated in Figure 4a, and the code disk outfitted with the system is displayed in Figure 4b [21]. The inner circle of the code disk in the intended system is incremental code that is equally carved around the circumference, whereas the outer circle of the code disk is 10-bit pseudo-random code. The code disk is matched with the shape and rotation angle of the detectors corresponding to the two code channels, which enables the detector array to gather as much optical signal as possible.

### 2.3. Readout Circuit Design

#### 2.3.1. Pseudo-Random Channel

The absolute code disc, which uses a pseudo-random code, is composed of a fixed length of engraved lines that are either translucent or opaque and positioned around the circumference of the measurement circle. The photoelectric receiving circuit corresponding to the pseudo-random code channel was designed based on the image sensor architecture, which is regarded as a “camera” to capture photos the pseudo-random code and obtain absolute position information in practical applications.

The main grating is illuminated by the LED light source during the normal functioning of the system. Because the pseudo-random code channel corresponds to the indicator grating in the full open window mode, the detector-corresponding readout circuit directly collects the light signal through the main grating. The light signal is subsequently converted to a current signal by the photodiode. In addition, the amplification circuit corresponding to each detector then amplifies the current and stores the charge on the cap. The pseudo-random signal readout principle is similar to the image sensor, in which the pixel circuit adopts the 3T structure. Furthermore, the integration and amplification of the photocurrent are realized by controlling the integration capacitor, with the read analog signal being finally transmitted to the outside of the chip.

#### 2.3.2. Incremental Channel

**Figure 5:**
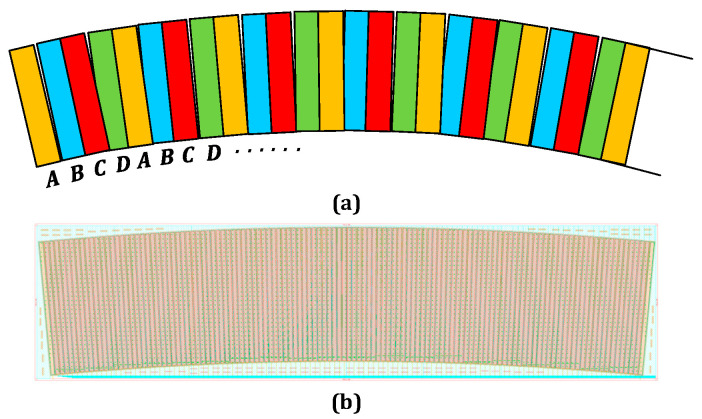
(**a**) Incremental channel detector arrangement. (**b**) Layout of the detector array.

The indication grating and the code plate displayed in Figure 5a produce four sets of Moiré stripe signals for the incremental code channel as they move in relation to one another, and the matching photodetector detects them. The detector array is developed as four interleaved groups, A, B, C, and D, primarily because the Moiré stripe signals comprise four sinusoidal signals with a 90° phase difference. A set of light and dark codes corresponds to each of the four detectors. Figure 5b depicts the layout of the detector array.

**Figure 6:**
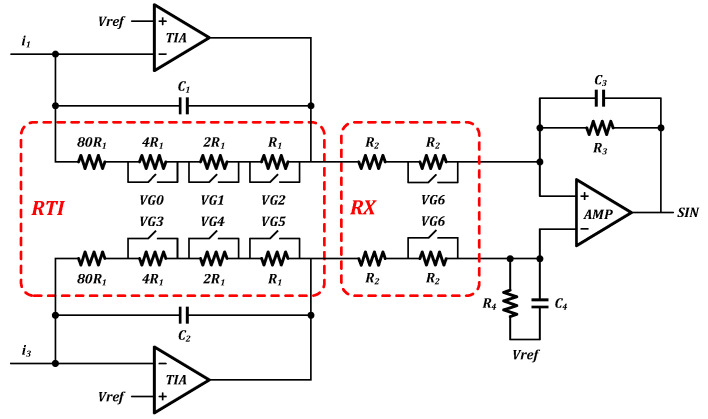
Architecture of the incremental code channel readout circuit.

Figure 6 depicts the overall design of the circuit for the incremental signal readout. More specifically, an amplifier circuit transforms the incremental photoelectric signal from the detector array, which has a phase difference of 90°, into a sinusoidal analog signal. A low noise, high gain transimpedance amplifier is utilized in the first stage of the amplifier to transform the nanoampere-level photocurrent into a millivolt-level voltage signal. Two sets of signals with a 180° phase difference are simultaneously processed by a differential amplifier to remove even harmonics and boost the signal amplitude before being output to a post-stage ADC converter. An adjustable resistor array module is installed at the front end of the amplifier to account for variations in actual light intensity. The gain of the amplifier is modified by adjusting the resistance of the resistor array using a control switch port that has been set aside for this purpose [22].

### 2.4. SAR ADC

A dedicated on-chip integrated analog-to-digital converter should be designed to quantize and subdivide the analog output signal of the incremental code channel, which is used to measure the angular displacement. Table 1 compares the performance of several common ADC architectures. In contrast to flash ADC, pipeline ADC, and sigma-delta ADC, SAR ADC have medium-to-high accuracy and sampling rate [23,24,25]. The proposed angular displacement sensor is primarily designed for system miniaturization and full integration; therefore, on the basis of satisfying the accuracy and sampling rate of the angular displacement sensing chip, this study selects a successive approximation ADC architecture that is more suitable for small-area monolithic integration and low power consumption.

**Figure 7:**
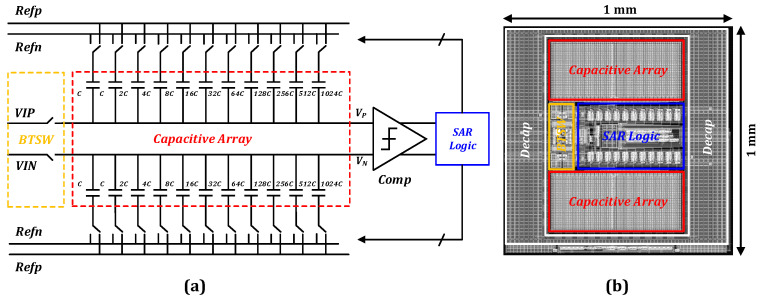
(**a**) Architecture of SAR ADC. (**b**) Layout of SAR ADC.

To interpolate the incremental code channel signal in this study, a SAR ADC with 12-bit resolution and 1MSPS sampling rate was developed. The architecture of the SAR ADC is depicted in Figure 7a, which also comprises modules for the comparator, bootstrapped switch, DAC array, and SAR logic. The layout of the SAR ADC is depicted in Figure 7b, with a layout area of 1 mm × 1 mm. To achieve a low sampling noise, the DAC capacitor array uses a standard binary capacitor array. The substrate and power supply noises were reduced by employing a fully differential structure. The performance of the SAR ADC is protected from nonlinear errors introduced by the standard sampling switch using a bootstrapped switch. The circuit uses synchronous sampling, and both comparator control clocks are generated using an external clock.

The sample and hold circuit of the developed SAR ADC utilizes a bootstrapped switch whose on-resistance is independent of the input signal to ensure the linearity of the ADC. The circuit architecture of the DAC array is illustrated in Figure 7a. The DAC module in the system utilizes a charge redistribution architecture. Moreover, the circuit uses a full differential design to reduce the substrate and power supply noise, which effectively reduces the common-mode noise. The capacitor network serves as both a sample and hold circuit and a DAC capacitor array in the charge-redistribution-based architecture.

**Figure 8:**
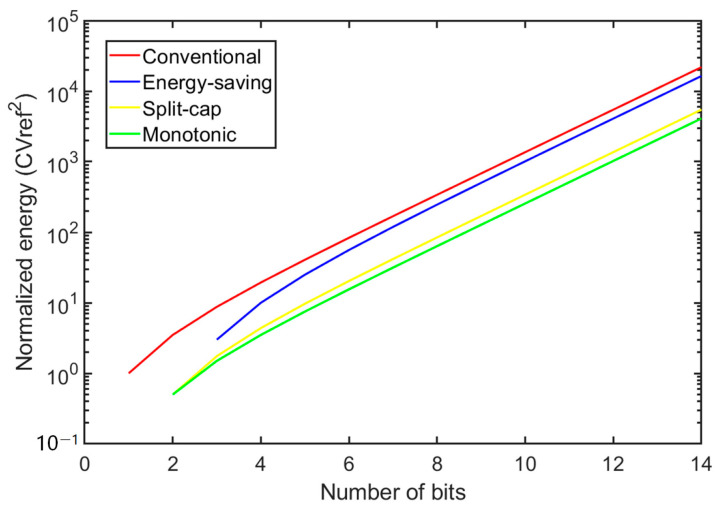
Comparison of the energy consumed by different switching algorithms.

The design used a monotonic switching algorithm to control the DAC capacitor array [16]. Compared with several common switching algorithms, the monotonic switching algorithm has the best energy efficiency for the same number of bits [26,27,28,29]. More specifically, the monotonic switching algorithm reduces the total sampled capacitance value by half using the top plate sampling method, reducing the layout area, and decreasing the DAC build time compared to conventional switching algorithms. Figure 8 demonstrates the comparison of the energy consumed by different switching algorithms. The energy consumed by the monotonic switching algorithm and conventional switching algorithm is

(2) $${E_{\mathrm{mono}}|}_{n=12}=\overset{n\;-\;1}{\underset{i=1}{\sum }}2^{n\;-\;2\;-\;i}{\mathrm{CV}}_{\mathrm{REF}}^{2}\;{=1024\mathrm{CV}}_{\mathrm{REF}}^{2}$$

(3) $${E_{\mathrm{conv}}|}_{n=12}=\sum_{i=1}^{n}2^{n+1\;-\;2i}(2i-{1)\mathrm{CV}}_{\mathrm{REF}}^{2}{=4551\mathrm{CV}}_{\mathrm{REF}}^{2}$$

Equations (1) and (2) reveal that when the accuracy is 12-bit, the monotonic switching algorithm can save 77% more energy than a conventional switching algorithm.

**Figure 9:**
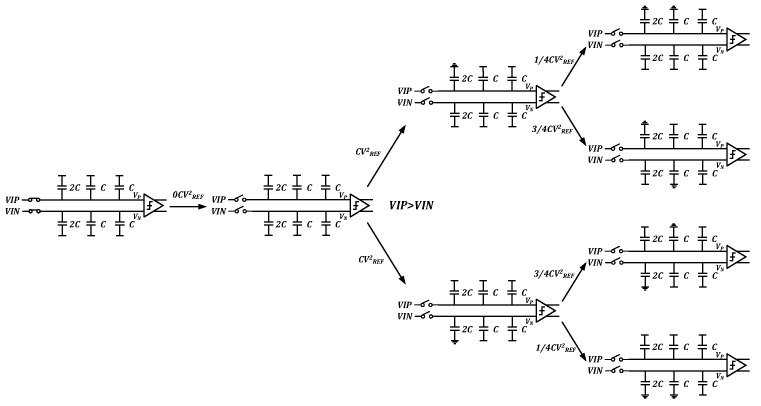
Diagram of 3-bit conversion process of the monotonic switching algorithm.

Figure 9 demonstrates that once the sampling switch is closed, the comparator immediately executes the first comparison without switching any capacitor or consuming energy, and only one capacitor is switched in the following switching steps based on the comparator comparison result. Moreover, it reduces power consumption and simplifies the digital circuitry used for logic control.

**Figure 10:**
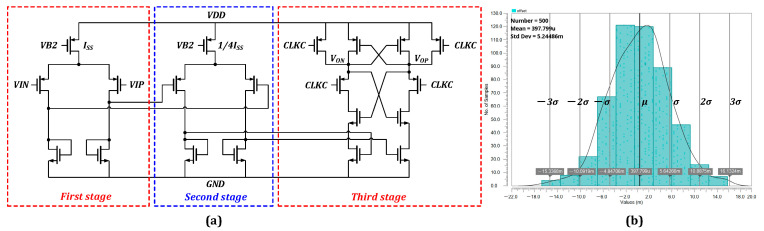
(**a**) Diagram of the comparator. (**b**) 500 times Monte Carlo simulation results for the comparator offset.

The performance of the SAR ADC was significantly affected by the comparison speed and comparator noise during the design stage. To reduce the power consumption of the SAR ADC and increase the sampling rate, as shown in Figure 10a, the comparator proposed in this study comprises a two-stage pre-amplification with a one-stage dynamic comparator. With the static pre-amplification of the first two stages, and the kickback noise and offset voltage of the third stage are attenuated by the gain of the first two stages, thereby achieving a lower comparison noise. The small input signal was pre-amplified simultaneously to increase the comparison speed. In the noise simulation, the input noise of the comparator was determined to be approximately 106 μV, which is below a quarter of the LSB, and can satisfy the operational requirement of a 12-bit ADC. In total, 500 Monte Carlo simulations of the offset of the comparator were executed in the present work, and the results are displayed in Figure 10b. The results demonstrate that the offset of dynamic comparator is approximately 397.79 μV, which is below half of the LSB.

## 3. Chip Implementation and Verification

### 3.1. Design Parameter

**Figure 11:**
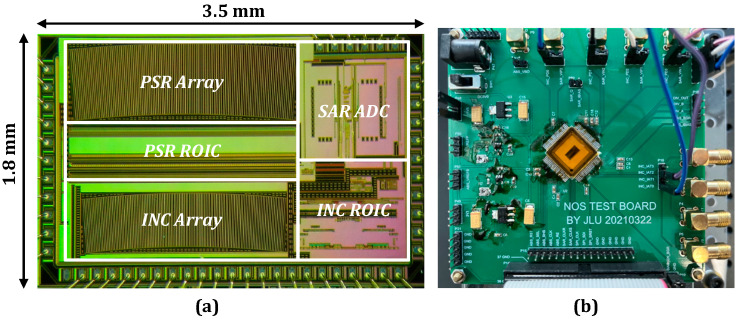
(**a**) Micrograph of the chip. (**b**) Test board.

Figure 11 illustrates the micrographs of the chip verification in the 0.35 μm standard CMOS process with an overall area of 3.5 × 1.8 mm^2^. For an angle of 7.03°, the detector array for the two code channels is arranged in a fan shape. On the one hand, this arrangement can guarantee the signal quality while preventing errors caused by the code disk contamination and uneven light source. However, the line array of pixels significantly increases the sensor speed. Table 2 illustrates the performance comparison of several typical ADC architectures.

### 3.2. Pseudo-Random Verification

The primary components of the optical system in the angular displacement sensing system include the light source, code disc, and indicator grating. The light source had a significant impact on the quality of the photoelectric signal. The light source of the proposed system comprises an IC-TL46 blue LED with a typical wavelength of 460 nm. The design of a high-linearity grating reading head with a light source divergence angle of 1°, and an effective spot diameter of 8 mm is possible owing to the shorter wavelength of the blue LED, which can also reduce the diffraction effect and increase the photoelectric conversion efficiency. To meet the need for high precision, the disk used in this study is a glass code plate with bright and dark code channels etched on the chrome-plated surface of the glass. Compared to other materials, the glass code plate has good temperature stability, thereby enhancing the capacity of the angular displacement sensor for adapting to various application conditions.

Verifying the consistency of the photoelectric response of the pixel array is the primary concern for the pseudo-random code channel. This study developed a test system based on the software and hardware co-design principle to evaluate the performance of the pseudo-random code channel under lighting conditions. The collected high-speed signals are transmitted to the host computer in real time via a gigabit ethernet, and the host computer uses LabVIEW 2015 to display the waveforms and save the data in real time.

**Figure 12:**
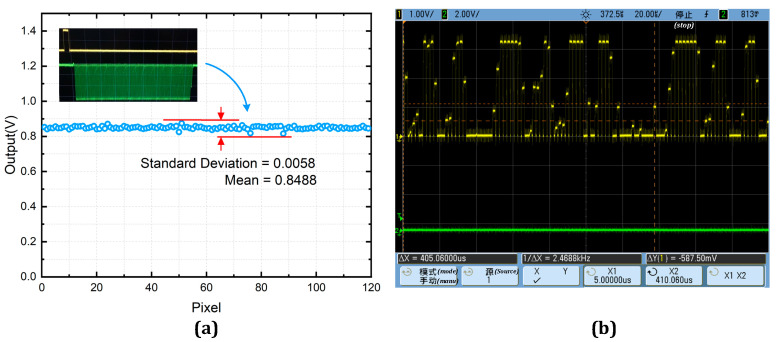
(**a**) Results of the optical consistency test. (**b**) Pseudo-random channel output signal.

Figure 12a illustrates the output voltage value of the picture element in the open window of the pseudo-random code channel for a single cycle, which demonstrates that the pseudo-random channel has a photoelectric consistency of over 93%. Accordingly, an optical encoder test prototype is developed, and the pseudo-random code channel is tested in combination with the designed optical code disc, and Figure 11b illustrates the output signal of the pseudo-random channel.

**Figure 13:**
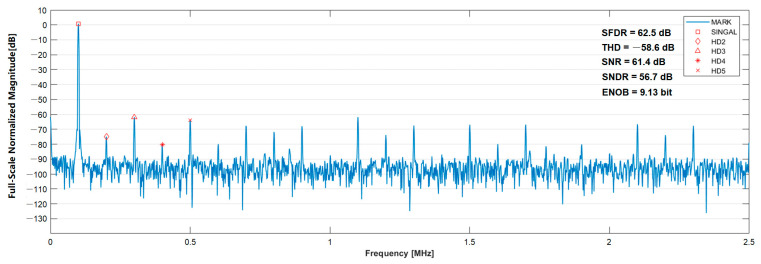
Test result of SAR ADC.

The incremental signal channels were verified using the built test system. The SAR ADC module was tested and verified. Figure 13 displays the results of the ADC dynamic performance test. The SAR ADC has an SNDR of 56.7 dB, SFDR of 62.5 dB, ENOB of 9.13 dB, and power consumption of 4.0 mW when the input signal is 100 kHz. The common mode voltage is 870 mV, and the sampling rate is 1 MHz.

**Figure 14:**
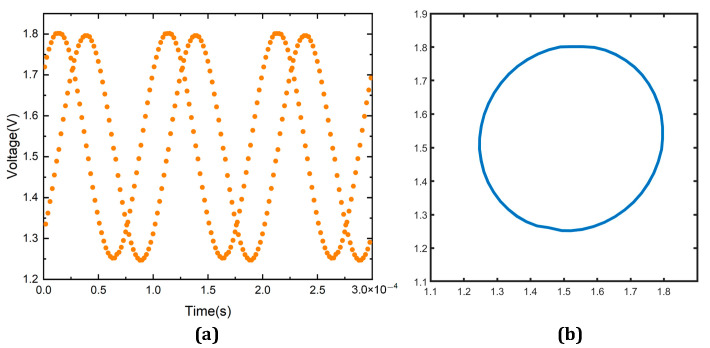
(**a**) Incremental channel output signal. (**b**) Lissajous circle.

The field-programmable gate array (FPGA) was configured to supply control signals for the chip to be tested. Subsequently, the sinusoidal voltage generated by the signal generator was employed to control the light and dark changes of the light source. The results displayed in Figure 14 demonstrate that even without considering the errors caused by the grating and axis system structure, the incremental channel can output a satisfactory signal.

A summary of the features of previously reported encoder systems isprovided in Table 3, where it is evident that they were designed mainly for separate detector chips or processing circuits [13,30]. The study of [13] describes a photodiode array chip for hybrid encoders. This is based on a CMOS process integrating four photodiode arrays that can read out M-codes and incremental codes in parallel, using off-chip processing circuitry amplifying the electrical signals and processing the data. According to [30], an area image-based optical encoder based on a CMOS image sensor was designed. The processing circuit for the received image data comprised a complex programmable logic device and a digital signal processor. The fully integrated angular displacement sensing chip proposed in this study uses the CMOS process to design the photodetector and processing circuit in one chip, as well as integrating the high-precision-data conversion circuit. This greatly reduces the complexity of the off-chip auxiliary circuit and enhances the integration of the optoelectronic angular displacement sensor. In contrast to the encoder reported by [16], the system used in this study is designed with a 12-bit SAR ADC, which completes the full function implementation of the angular displacement sensing system on a very small chip size having a lower power consumption at a higher system resolution.

## 4. Conclusions

A fully integrated line-array-type absolute angular encoder position-sensing chip is proposed to read out position data using hybrid coding, and mainly includes pseudo-random code channel detector, incremental code channel detector, a readout circuit corresponding to the readout code channel, and on-chip SAR ADC. It is characterized by the design of detector arrays that match the code disc and the optimization of the detector shape and angle according to the curvature of the code disc to allow the system to realize better high-speed weak light detection. The incremental code channel readout circuit was designed based on the transimpedance amplifier structure, while the amplifier gain can be adjusted by configuring the module. A fully differential 12-bit, 1MSPS sampling rate SAR ADC based on the charge redistribution principle was designed for the quantization and signal interpolation of the incremental code channel output signal. In addition, it was integrated on-chip with detector arrays and readout circuits by a 0.35 μm CMOS process. The performance of the design circuit was validated by performing experimental measurements. Furthermore, the photoelectric consistency of the output signal of the pseudo-random code channel was >93%, with the measured ENOB of the SAR ADC being 9.13 bit while the incremental code channel could output a more desirable Lissajous circle. Finally, the area of the overall system used has a dimension of 3.5 mm × 1.8 mm.

## Figures and Tables

**Table 1:** Performance comparison of several typical ADC architectures.

Type	Precision	Speed	Power Consumption	Area
SAR	Higher	Medium	Low	Small
Flash	Low	Ultra-fast	Ultra-high	Ultra-large
Pipeline	Medium	Fast	Medium	Large
Sigma-Delta	Ultra-high	Slow	Higher	Larger

**Table 2:** Main Parameters of the Chip.

Parameter	Value	Parameter	Value
Technology	0.35-μm CMOS	Size	3.5 × 1.8 mm^2^
Pitch	25 μm	Number of detectors	120 × 2
Supply	3.3 V	Power consumption	29 mW
Frame	7400 frame/s	Consistency of output	>90%

**Table 3:** Comparison of key features.

	Literature [16]	Literature [13]	Literature [30]	This Paper
Array	Area	Area	Area	Linear
Technology	0.6 μm CMOS	0.35 μm CMOS	/	0.35 μm CMOS
ADC on-chip	8-bit SAR	None	None	12-bit SAR
Chip-Size	4 mm × 4 mm	5.5 mm × 3.0 mm	4 mm × 3 mm	3.8 mm × 1.5 mm
Power Consumption	75 mW	/	/	29 mW
Resolution	14-bit	11-bit	12-bit	22-bit

## Data Availability

Data underlying the results presented in this paper are not publicly available at this time but may be obtained from the authors upon reasonable request.
